# Stagewise oxygenation of the Earth’s atmosphere and oceans

**DOI:** 10.1016/j.xinn.2026.101353

**Published:** 2026-03-17

**Authors:** Haiyang Wang, Chao Li

**Affiliations:** 1 State Key Laboratory of Oil and Gas Reservoir Geology and Exploitation & Institute of Sedimentary Geology, Chengdu University of Technology, Chengdu 610059, China; 2 International Center for Sedimentary Geochemistry and Biogeochemistry Research, Chengdu University of Technology, Chengdu 610059, China; 3 Key Laboratory of Deep-time Geography and Environment Reconstruction and Applications of Ministry of Natural Resources, Chengdu University of Technology, Chengdu 610059, China

**Keywords:** atmospheric oxygen levels, Great Oxidation Event, triple-oxygen isotopes, oxygen oases, oceanic oxygenation

## Abstract

Earth’s surface oxygenation was a protracted, multi-step process. It is widely accepted that atmospheric O_2_ rose through three major steps, each closely linked to key evolutionary innovations. Recent advances, particularly in sulfate triple-oxygen isotope records, now provide new constraints on the timing, tempo, and magnitude of atmospheric oxygenation. These data reveal a positive coupling between atmospheric and oceanic oxygenation on billion-year timescales, with stagewise rises in atmospheric O_2_ consistently preceding oceanic responses, whereas on shorter, million-year timescales, their redox states could diverge, exhibiting negative coupling. Oceanic redox evolution was also spatially and temporally heterogeneous, progressing from localized Archean oxygen oases (>3.0 Ga) to a globally oxygenated state by the Paleozoic (<0.41 Ga). Here, we synthesize geochemical, sedimentological, and palaeobiological evidence to reconstruct this coupled and dynamic atmosphere-ocean oxygenation history and to reassess its timing, mechanisms, and ecological consequences. These deep-time perspectives illuminate the co-evolution of life and Earth’s surface environments and provide a conceptual framework for evaluating planetary habitability.

## Public summary

• Earth’s O_2_ rose unevenly: shallow seas first, then the atmosphere, and much later the deep ocean.
• Triple-oxygen isotopes reveal three major stages in atmospheric O_2_ rise.
• During a 2-billion-year transition, atmosphere and ocean O_2_ sometimes diverged despite long-term rise.
• Ocean oxygenation was highly variable, with extreme spatial heterogeneity over time.

## Graphical abstract

**Figure:**
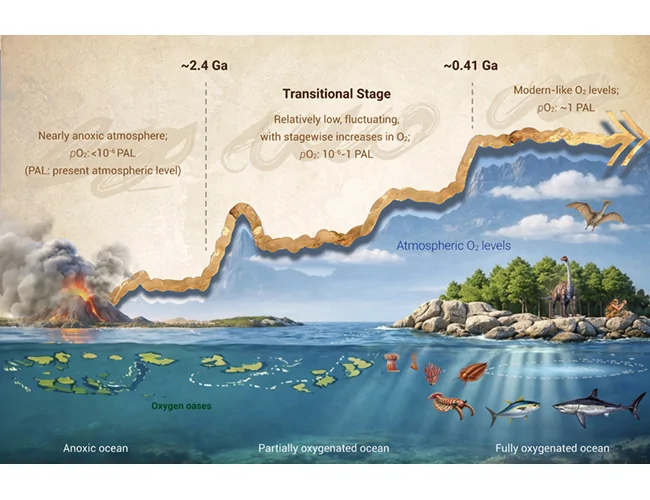

## Introduction

Earth remains the only known planet in the universe capable of sustaining complex life. A defining feature of its habitability is the presence of an oxygen-rich atmosphere (∼21% O_2_ by volume) combined with well-oxygenated oceans, which together support the respiration of diverse aerobic organisms. Yet, early in Earth’s history, its surface was entirely anoxic. The rise and later fluctuation of oxygen profoundly reshaped Earth’s geochemical and energy cycles, modulated climate, and guided the evolutionary trajectory of life. Consequently, reconstructing Earth’s oxygenation history is central to understanding how it became and remained habitable.

Recent advances in geochemical proxies, most notably sulfate triple-oxygen isotopes (Δ′^17^O), provide new constraints on atmospheric oxygenation, complementing and in some cases revising interpretations from traditional ocean redox proxies. Sedimentary records further reveal that ocean oxygenation was delayed, spatially heterogeneous, and punctuated by multiple transitions before achieving its modern oxygenated state. This review synthesizes these developments with two aims: (1) to chart the rise of atmospheric O_2_ from absence to present levels, highlighting insights from sulfate Δ′^17^O and (2) to assess the timing, mechanisms, and consequences of ocean oxygenation, with emphasis on the interplay between localized oxygen oases and global atmosphere-driven processes. Integrating these perspectives clarifies the coupled evolution of the atmosphere, oceans, and life through deep time.

## Atmosphere-ocean transitional oxygenation framework

**Figure 1:**
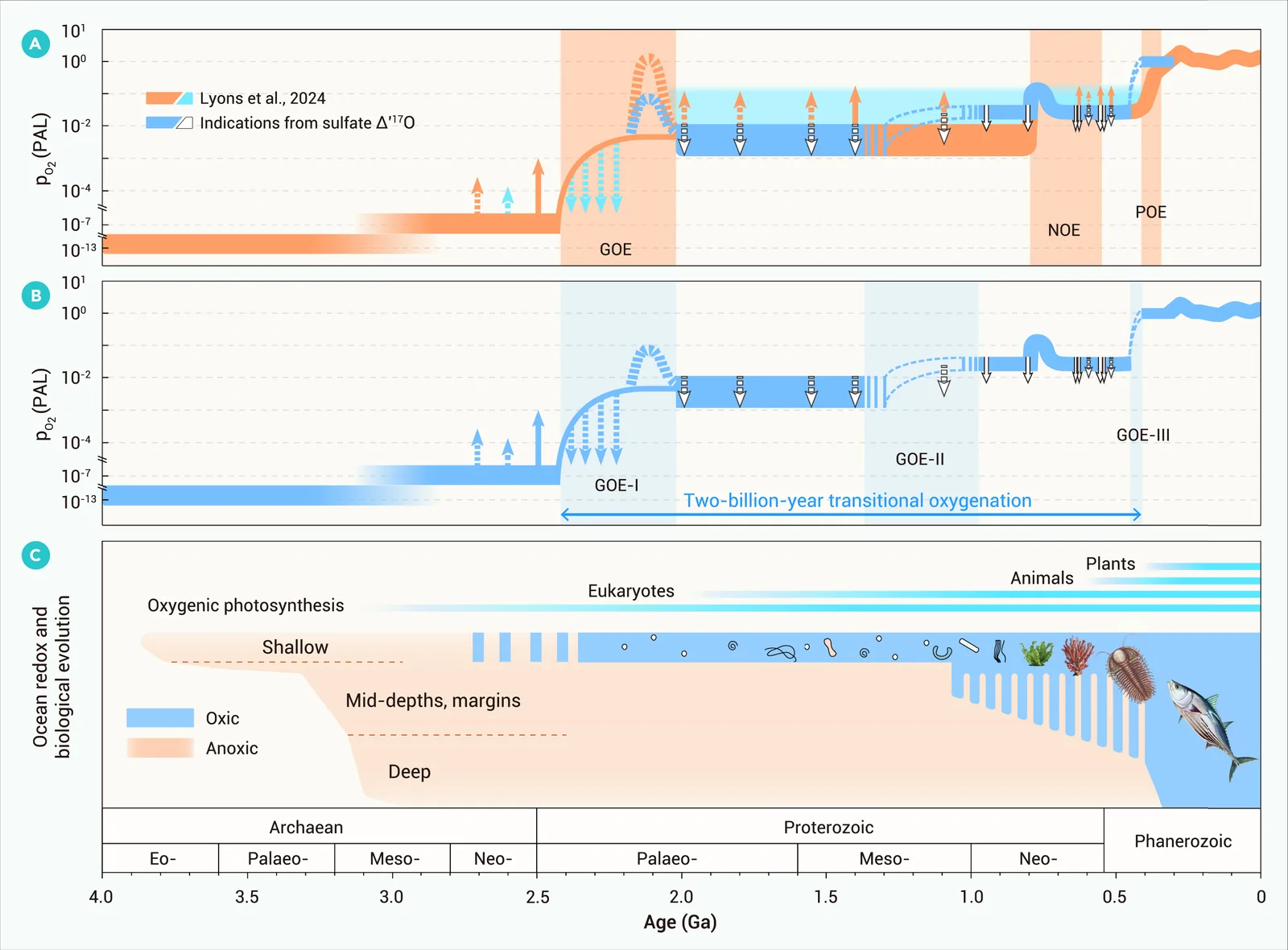
History of Earth’s atmospheric-oceanic oxygenation and biological innovation (A) Atmospheric O_2_ evolution inferred from previous studies,^1^ refined with compiled sulfate triple-oxygen isotope constraints.^3^ (B) Revised atmospheric *p*O_2_ history and atmosphere-centered GOEs proposed in this study. (C) Coupled evolution of oceanic redox conditions and biological innovation (modified from Wang et al.^3^). White dotted downward arrows indicate speculative declines in atmospheric O_2_, whereas white solid arrows denote decreases in atmospheric O_2_ documented during oceanic oxygenation events reported from Wang et al.^3^ GOE, Great Oxidation Event; NOE, Neoproterozoic Oxygenation Event; POE, Paleozoic Oxygenation Event.

It is widely accepted that Earth’s surface oxygenation unfolded in three major steps, each marking a pivotal threshold in atmospheric oxygen levels (*p*O_2_) and biospheric evolution (Figure 1).^1^^,^^2^^,^^3^ The first, the Great Oxidation Event (GOE, ∼2.4–2.1 Ga), transformed Earth from an essentially anoxic planet to one with persistently oxygenic atmosphere. Prior to this transition, *p*O_2_ likely remained below 10^−6^ of the present atmospheric level (PAL),^4^ as indicated by mass-independent sulfur isotope (Δ^33^S) anomalies.^5^ During the GOE, atmospheric O_2_ rose substantially, perhaps to 10^−3^–10^−1^ PAL,^1^ establishing the foundation for aerobic metabolisms and the early diversification of eukaryotic life.^2^^,^^6^^,^^7^ The second major step, the Neoproterozoic Oxygenation Event (NOE, classically ∼800–500 Ma)^8^ witnessed that *p*O_2_ may have exceeded 30% PAL at times, but with pronounced fluctuations.^9^ Periodic pulses of ocean oxygenation coincided with the radiation of multicellular algae and the earliest animals,^7^^,^^10^^,^^11^^,^^12^ underscoring the role of oxygen in enabling complex multicellularity. The third step, the Paleozoic (or Devonian) Oxygenation Event (POE/DOE, ∼419–359 Ma), marked a rapid transition to near-modern *p*O_2_ levels and widespread deep-ocean ventilation.^13^^,^^14^^,^^15^^,^^16^^,^^17^^,^^18^^,^^19^^,^^20^^,^^21^ This event supported the expansion of complex ecosystems and coincided with the colonization of land by plants.^22^ By enhancing organic carbon burial as well as weathering intensity on land, terrestrial vegetation could have fundamentally reshaped global redox cycles in the surface environments.^23^^,^^24^ Collectively, these oxygenation steps established the environmental template for the flourishing of complex life.

Although linked, atmospheric and oceanic oxygenation unfolded on different timescales. Following the GOE, O_2_ remained restricted to surface waters, while the deep ocean persisted in an anoxic state until broadly ventilated after the POE (Figure 1C).^13^^,^^14^^,^^15^^,^^16^^,^^17^^,^^18^ This prolonged delay reflects both limited atmospheric O_2_ supply and the vast reservoirs of reduced species (Fe^2+^, Mn^2+^, HS^−^/H_2_S, organics) that buffered oxygen penetration into the ocean interior.^25^^,^^26^^,^^27^^,^^28^^,^^29^^,^^30^^,^^31^ Even after the final establishment of high atmospheric *p*O_2_ and well-ventilated oceans, episodic oceanic anoxic events recurred, underscoring the strong influence of local and regional processes on water-column oxygen levels.^32^^,^^33^^,^^34^^,^^35^ Together, these patterns highlight the asynchronous and spatially heterogeneous nature of Earth’s surface oxygenation.

## New insights from sulfate Δ′^17^O for atmospheric O_2_ evolution

The application of sulfate Δ′^17^O as a direct tracer of ancient atmospheric O_2_ marks a breakthrough in reconstructing Earth’s surface oxygen evolution.^3^^,^^12^^,^^36^^,^^37^^,^^38^ Atmospheric O_2_ carries a characteristic negative ^17^O anomaly, generated by O_2_-O_3_-CO_2_ photochemical reactions, which can be transferred into sulfate during sulfide oxidation. As a result, sedimentary sulfate Δ′^17^O provides an archive more directly tied to past atmospheric chemistry,^39^^,^^40^ offering a clearer signal than conventional marine redox proxies. For further insights into the sulfate Δ′^17^O proxy for atmospheric O_2_ levels refer to supplemental information.

A central outcome of this approach is a refined chronology of atmospheric oxygenation (Figures 1A and 1B). The sulfate Δ′^17^O framework places the GOE at ∼2.4–2.1 Ga, in agreement with previous constraints, but indicates that subsequent rises in atmospheric O_2_ occurred earlier than traditionally inferred. Specifically, sulfate Δ′^17^O data suggest that the atmospheric “NOE” may have begun before 1.0 Ga, in the Mesoproterozoic, and that the Paleozoic rise in atmospheric O_2_ likely occurred between ∼440 and 410 Ma,^3^ more than about 200 and 30 Myr earlier than the canonical timings of the NOE and POE, respectively. This mismatch arises largely because earlier proxies—such as sulfate evaporites,^41^ Mo-U-Se-Tl isotopes,^14^^,^^20^^,^^21^^,^^42^^,^^43^^,^^44^ trace element ratios,^8^^,^^15^^,^^45^^,^^46^^,^^47^^,^^48^^,^^49^^,^^50^ and Fe^3+^/ΣFe in marine basalts^16^—primarily reflect ocean chemistry rather than atmospheric composition. As such, they provide only indirect and often variable constraints on atmospheric oxygenation. More broadly, ocean redox is not always tightly coupled to atmospheric *p*O_2_, being strongly influenced by local nutrient supply, biological productivity, circulation, and basin geography.^51^^,^^52^^,^^53^^,^^54^

By contrast, the proxies that are more directly tied to atmospheric conditions align well with the sulfate Δ′^17^O framework. Chromium isotopes, reflecting oxidative weathering of manganese on land, suggest a rise in atmospheric *p*O_2_ by ∼1.35 Ga.^55^ Sedimentary charcoal, which requires *p*O_2_ above ∼75% PAL to sustain wildfire, indicates that such levels were reached by ∼420 Ma.^18^^,^^19^ Notably, both Δ′^17^O and Δ^33^S—the two most direct atmospheric O_2_ proxies—converge on the GOE as the first sustained oxygenation step, underscoring the robustness of an atmosphere-first view (Figures 1A and 1B).^3^ From above constraints, atmospheric oxygenation seems to always precede oceanic oxygenation during each major step, a sequence that is physically plausible: atmospheric O_2_ can rise relatively rapidly once oxygen sources exceed its sinks (particularly through oxidative weathering on land),^56^ whereas oceanic oxygenation is hampered by solubility limits, water-column stratifications, and vast reductant reservoirs (Fe^2+^, Mn^2+^, HS^−^/H_2_S, organics).^25^^,^^26^^,^^27^^,^^28^^,^^29^^,^^30^^,^^31^

A further implication of sulfate Δ′^17^O record is the recognition of a complex atmosphere-ocean coupling.^3^^,^^38^ Between ∼1,000 and 410 Ma, repeated negative carbonate carbon-isotope (δ^13^C) excursions likely reflect oceanic oxygenation pulses.^48^^,^^57^ Coupled shifts in sulfate-Δ′^17^O, sulfate-δ^34^S, and carbonate-δ^13^C, together with biogeochemical modeling, indicate that these oxygenation events arose episodically due to increased atmospheric O_2_ influx, which growingly oxidized reduced carbon and sulfur pools in the oceans and produced the covarying C-S-O isotope signals.^3^^,^^12^ Although these processes elevated oceanic oxidation state, they also lowered atmospheric *p*O_2_ temporarily due to rapid oxygen consumption in a million-year timescale.^3^^,^^9^ The emerging Earth’s oxygenation history thus reflects a dual pattern of atmosphere-ocean interaction: long-term positive coupling (a billion-year trajectory toward higher O_2_ levels in both reservoirs) overprinted by short-term negative coupling (million-year pulses in which oceanic oxidation drew down atmospheric *p*O_2_). This finding challenges the long-standing assumption that transient oceanic oxygenation events indicate short-term rises in atmospheric *p*O_2_, often depicted as upward arrows in the O_2_ evolution curves of the atmosphere (Figure 1A). Instead, each transient oceanic oxygenation pulse could reflect enhanced process of siphoning O_2_ from the atmosphere into the oceans, followed by rapidly consumed by abundant reductants within. Consequently, many such events may reflect temporal atmospheric O_2_ declines and should be represented with downward arrows (Figures 1A and 1B). These negative-feedback processes slowed the stepwise climb of atmospheric O_2_, making its rise arduous and uneven, while simultaneously driving progressive oceanic oxygenation. Ultimately, between ∼410 and ∼380 Ma, the two reservoirs converged to reach near-modern oxygen levels (Figures 1B and 1C).^3^^,^^13^^,^^14^^,^^15^^,^^16^^,^^17^^,^^20^^,^^21^^,^^38^

Taken together, the sulfate Δ′^17^O perspective invites a rethinking of how we define and name Earth’s oxygenation events. A nomenclature emphasizing atmospheric thresholds (e.g., GOE-I, GOE-II, GOE-III) explicitly reflects the atmosphere-first sequence and helps reconcile discrepancies between atmosphere- and ocean-based proxies. This framework preserves continuity with earlier terminology while underscoring the primacy of atmospheric changes in Earth’s surface oxidation history. GOE-I (2.4–2.1 Ga), GOE-II (1.3–1.0 Ga), and GOE-III (0.44–0.41 Ga) primarily reflects three major rises in atmospheric *p*O_2_, whereas the traditionally defined NOE (∼0.8–0.5 Ga) and POE (∼0.42–0.36 Ga) mainly represent important episodes of oceanic oxygenation that responded to the latter two atmospheric O_2_ increases. Hereafter, the terms GOE-I/-II/-III refer to atmospheric oxygenation, whereas NOE and POE denote oceanic oxygenation. Notably, GOE-I marked the overcoming of O_2_ sinks significantly from oxidative weathering of large reduced crustal reservoirs on land, whereas GOE-II/NOE and subsequent GOE-III/POE substantially involved the oxidation of extensive reduced carbon, sulfur, and other pools within the oceans. Consequently, each oxygenation step was protracted, unfolding over several hundred million years (Figure 1B).

Here, we integrate sulfate Δ′^17^O records with earlier geochemical proxies and biogeochemical modeling to provide the most refined constraints to date on Earth’s atmospheric O_2_ trajectory (Figure 1B). The timing of three stepwise rises in atmospheric O_2_ is delineated by three major shifts in sulfate Δ′^17^O dataset.^3^ These data further indicate that the putative atmospheric O_2_ “overshoot” during the Lomagundi event (∼2.3–2.1 Ga; the largest positive carbon-isotope excursion in Earth history) was more limited than previously inferred. Sulfate Δ′^17^O values during this interval are consistently more negative than modern values, yet comparable with those of the Neoproterozoic-Paleozoic, implying that atmospheric O_2_ did not reach near-modern levels despite enhanced organic carbon burial.^58^ Biogeochemical Earth system modeling calibrated with sulfate Δ′^17^O further suggests persistently low atmospheric O_2_ levels of ∼0.1%–1% PAL throughout much of the mid-Proterozoic (∼2.0–1.4 Ga).^3^^,^^36^ Finally, sulfate Δ′^17^O records point to a substantial rise in atmospheric O_2_ between ∼1.4 and 1.0 Ga, exceeding ∼1% PAL; however, absolute *p*O_2_ thereafter remain poorly constrained. We therefore retain previous atmospheric O_2_ estimates for this interval until the third major rise at ∼0.44–0.41 Ga, when *p*O_2_ approached near-modern levels.^3^^,^^37^^,^^38^ A caveat is that additional stepwise O_2_ rises beyond GOE-II during the ∼2.4–0.41 Ga transition may have gone undetected due to limited sulfate Δ′^17^O resolution. The traditional GOE-NOE-POE framework, implying three discrete oxygenation steps, is therefore likely oversimplified. We instead adopt a cautious three-stage model^3^ that allows multiple unresolved stepwise increases within this 2-billion-year transition, pending higher-resolution data.

## Patchy oxygen oases and pulsed atmosphere-ocean oxygenation

We further explore the processes and mechanisms that governed oceanic oxygenation from its earliest stages (>3.0 Ga) to the present. Oceanic oxygenation reflects the balance between oxygen sources and sinks. Oxygen enters water mainly through two pathways: local production by oxygenic photosynthesis and global atmospheric influx via air-sea exchange. These endmember processes define two contrasting yet coexisting regimes, the Local Production Oxygen Mode (LPOM) and the Global Atmospheric Oxygen Mode (GAOM). Although most environments represent a mixture of both, distinguishing them helps clarify the mechanisms, spatial scales, and consequences of oceanic oxygenation event.

In the LPOM scenario, oxygen accumulation is driven primarily by *in situ* photosynthesis. When local oxygen production outpaced the consumption by reduced chemical species, oxygen-rich zones, that is oxygen oases, developed, with dissolved oxygen concentrations higher than in surrounding low-oxygen or anoxic waters. Such oxygen oases were likely restricted to nearshore, sunlit settings where light, nutrients, and ecological stability favored oxygen production. Modern analogs, such as anoxic lakes in polar regions, show how local photosynthesis can generate benthic micro-oxic zones within otherwise oxygen-free systems.^59^^,^^60^ By contrast, the GAOM scenario reflects large-scale oceanic oxygenation facilitated by atmospheric O_2_ influx. In this scenario, oxygen dissolves from the atmosphere into surface waters and is redistributed vertically and horizontally through mixing, overturn, and thermohaline circulation.^51^ Semi-enclosed seas such as the modern Baltic and North Seas illustrate this process: winter mixing restores deep-water oxygen following summer stratification and organic matter-driven hypoxia.^61^^,^^62^ The efficiency of GAOM depends on basin morphology, temperature gradients, and salinity stratification, which together regulate O_2_ transport into the ocean interior.

LPOM and GAOM differ in mechanism, spatial extent, and ecological impact. LPOM operated in patchy nearshore settings, while GAOM reflects more pervasive oxygenation driven by atmospheric O_2_ and ocean circulation. These two regimes were not mutually exclusive: early Earth’s oceans were likely dominated by LPOM-style oases but, as atmospheric *p*O_2_ rose, GAOM became increasingly influential in the oceans. The dynamic transition and interaction between the two modes help explain why oceanic oxygenation advanced episodically and heterogeneously, amid a long-term rise in atmospheric *p*O_2_. Their interplay underpinned the progressive oxygenation of the oceans and the emergence of habitats that supported complex life.

**Figure 2:**
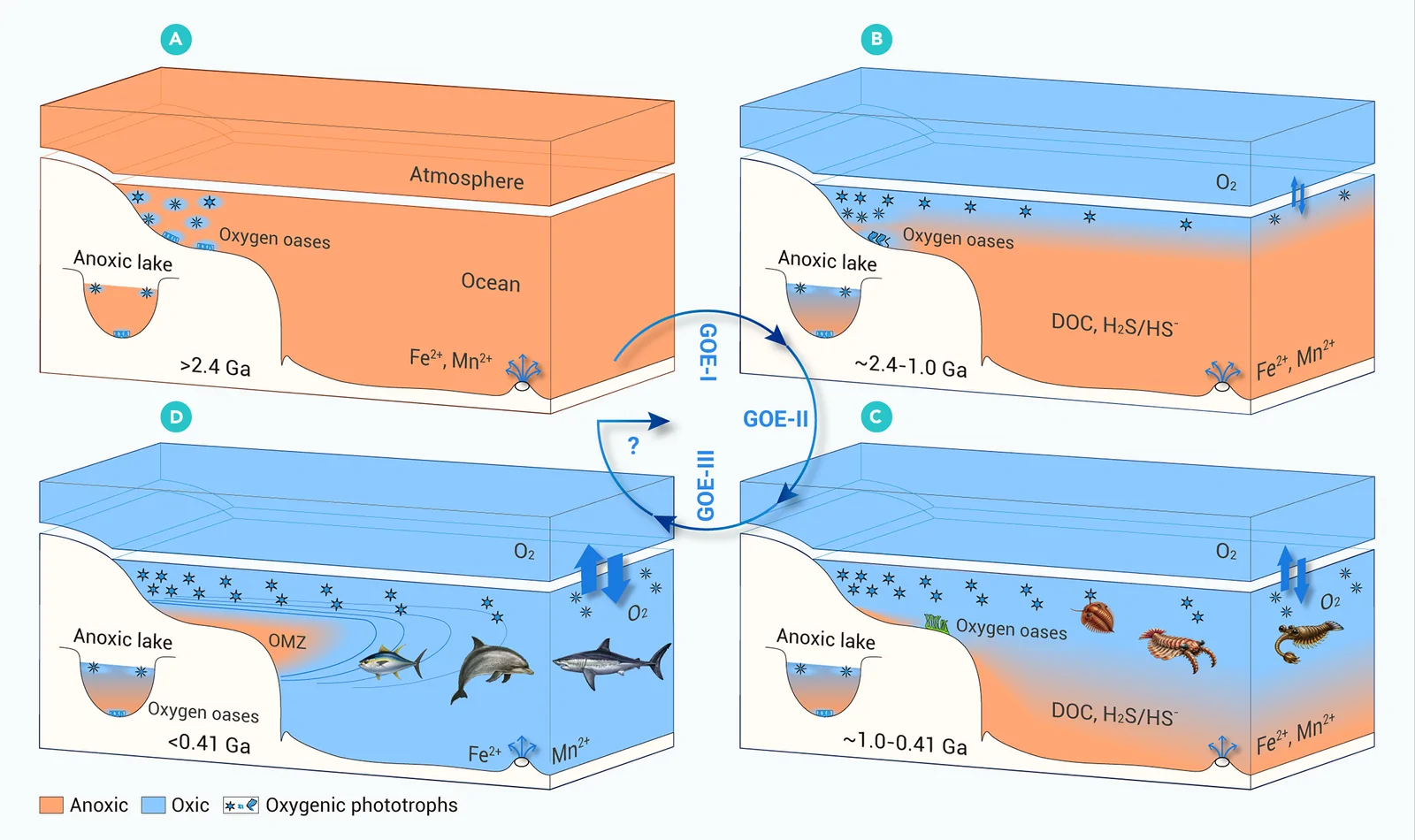
Evolution of Earth’s atmosphere-ocean redox states through time and space (A) Before 2.4 Ga: atmosphere and oceans were largely anoxic, with oxygen restricted to localized, shallow-water oxygen oases. (B) From 2.4 to 1.0 Ga: atmospheric O_2_ remained low; oxygenated waters were confined to the ocean surface, and oxygen oases developed primarily along continental shelves. (C) Between 1.0 and 0.41 Ga: atmospheric O_2_ increased but fluctuated, while oxygenated waters progressively penetrated to greater depths, with shelf environments continuing to host oxygen oases. (D) Since ∼0.41 Ga: atmospheric O_2_ approached near-modern levels; deep oceans became pervasively oxygenated, whereas mid-depth waters developed oxygen minimum zones (OMZs) that waxed and waned (solid curves) in response to high organic matter export and oxygen consumption. DOC, dissolved organic carbon. Note: sedimentary evidence for ancient terrestrial lakes is limited; these features are shown to illustrate potential redox heterogeneity rather than confirmed occurrences.

The earliest ocean oxygenation manifested as localized oxygen oases (Figure 2A), phototrophic niches in coastal zones, during the Archean, since oxygenic photosynthesis by early cyanobacteria began producing O_2_.^63^^,^^64^ Multiple geochemical lines of evidence, including iron speciation, trace-metal enrichments, rare earth element patterns, and Fe-Mo-Tl-S-N isotopic signatures, record brief, intermittent development of oxygenic conditions in shallow marine settings well before the GOE-I.^64^^,^^65^^,^^66^^,^^67^^,^^68^^,^^69^^,^^70^^,^^71^^,^^72^^,^^73^^,^^74^^,^^75^^,^^76^ At that time, however, Earth’s surface was dominated by vast reservoirs of reduced species and by reducing volcanic and metamorphic fluids that rapidly consumed liberated O_2_, preventing its spread beyond production sites.^25^^,^^26^^,^^56^^,^^77^ Nonetheless, model estimates suggest that dissolved oxygen concentrations in these oases could have reached >1–10 μM,^69^^,^^78^ sufficient to sustain aerobic metabolisms and alter nutrient cycles.^74^^,^^79^ The scale, duration, and impact of these oases were controlled by nutrient availability, particularly phosphorus, which limited marine productivity.^66^^,^^75^ Conceptually, early oxygen oases may have evolved through three stages: an incipient phase (>2.8 Ga) with short-lived, localized oxic patches^64^^,^^66^^,^^67^^,^^69^^,^^75^; an expansion phase (∼2.8–2.4 Ga) marked by more persistent oxygen oases promoted by possibly nutrient flux changes^65^^,^^68^^,^^70^^,^^71^^,^^72^^,^^73^^,^^74^^,^^76^; and a breakthrough phase (<2.4 Ga) when cumulative oxygen export from these oases began to overwhelm Earth’s reduced surface sinks, allowing atmospheric O_2_ to rise and initiating global-scale land and oceanic surface oxidation that defines the GOE-I.^77^^,^^80^^,^^81^

The GOE-I initiated a regime in which atmospheric O_2_ began exerting broader control over ocean redox conditions.^1^^,^^3^^,^^81^ By ∼2.3 Ga, coupled evidence from Δ^33^S and Tl isotopes indicates the first large-scale transfer of oxygen from the atmosphere to the oceans, marking the onset of a global atmosphere-driven oxygenation regime.^80^ Yet, throughout the following billion-year interval—the mid-Proterozoic preceding the GOE-II—dissolved oxygen levels in the surface waters remained generally very low,^30^^,^^82^^,^^83^^,^^84^ and water columns largely anoxic,^29^^,^^30^ as atmospheric *p*O_2_ likely fluctuated around 0.1%–1% PAL,^1^^,^^3^^,^^36^ insufficient for deep-water ventilation (Figures 1B and 1C). Under such low *p*O_2_, ocean oxygenation depended strongly on local photosynthetic activity. Modeling suggests that below ∼2.5%–10% PAL, *in situ* biological oxygen production can dominate over atmospheric O_2_ diffusion.^53^ Accordingly, the mid-Proterozoic ocean is best envisioned as a dynamic redox landscape—low-oxygen and/or anoxic water columns punctuated by shallow oxygen oases sustained by oxygenic phototrophs linked to episodic nutrient delivery, set against a backdrop of persistently low surface-water oxygen levels^54^ (Figure 2B).

Distinguishing oxygen oases sustained by local photosynthesis from oceanic oxygenation events driven by transient atmospheric O_2_ increases after the GOE-I is, however, more challenging. Before the GOE-I, under a strictly anoxic atmosphere, local photosynthesis was the only plausible source of ocean oxygenation. Afterward, the presence of a low but nonzero atmospheric O_2_ background complicates this distinction. Integrated sedimentological and geochemical evidence nevertheless provides important constraints. For instance, sedimentary bubble fabrics associated with microbial mats (1.6–1.4 Ga) indicate *in situ* oxygenic photosynthesis and local oxygen release,^85^ whereas pyrite sulfur isotopes combined with diffusion modeling suggest oxygenated seafloors within otherwise anoxic oceans.^86^ Shelf-to-basin redox data reveal pronounced spatiotemporal heterogeneity linked to nutrient availability, with phosphorus-rich intervals exhibiting enhanced oxygenation and thus implying locally elevated oxygen production.^52^ Mineralogical and I/(Ca+Mg) data indicate that microbial mats alone had limited oxygen-producing capacity, and that broader shallow-water oxygenation at ∼1.4 Ga likely reflected increased atmospheric O_2_ influx.^87^ More recently, however, Proterozoic I/(Ca+Mg) records show a latitudinal pattern of decreasing oxygen concentrations from the Equator to the mid-latitudes—opposite to the modern ocean—suggesting a biosphere-controlled rather than atmosphere-controlled oxygen distribution in the upper ocean.^54^ Taken together, these findings depict the mid-Proterozoic ocean as patchily oxygenated, with atmospheric O_2_ exerting growing influence while local processes remained vital (Figure 2B).

## Widespread atmosphere-ocean oxygenation and the rise of a well-oxygenated ocean

The second major rise in atmospheric O_2_ levels (GOE-II; Figure 1B) was nonlinear and accompanied—or closely followed—by large perturbations in the carbon, phosphorus, sulfur, and oxygen cycles of the Neoproterozoic oceans.^12^^,^^88^^,^^89^^,^^90^^,^^91^ Multiple atmosphere-ocean oxygenation events during this interval are recorded by diverse geological evidence, including thick sulfate evaporite accumulations,^41^ Cr-U-Fe isotopes,^83^^,^^92^^,^^93^^,^^94^ enrichments in redox-sensitive trace elements (Mo, U, V, Re, Cr, etc.),^8^^,^^47^^,^^49^^,^^50^ I/(Ca+Mg) ratios,^48^ Zn/Fe ratios,^46^ sulfate Δ′^17^O anomalies,^3^^,^^12^ and pyrite δ^34^S-Δ^33^S signatures.^95^ The Shuram Excursion—a globally synchronous, negative carbonate δ^13^C anomaly at ∼570 Ma and the largest in Earth’s history—exemplifies these processes. It reflects a global oceanic oxygenation event driven by increased atmospheric O_2_ influx, evidenced by coupled carbon, sulfur, and oxygen isotope variations^12^ and elevated carbonate-associated phosphate contents.^88^ Specifically, enhanced oxidation of a large dissolved organic carbon pool released ^13^C-depleted carbon and nutrient phosphorus, while concurrent sulfur oxidation transferred ^34^S-depleted sulfur and ^17^O-depleted atmospheric oxygen into marine sulfate.^12^^,^^96^ A recent study suggested similar mechanisms for other Neoproterozoic-Cambrian large negative carbonate δ^13^C excursions,^3^ supporting repeated cycles of organic carbon accumulation and oxidation linked to atmospheric-oceanic oxygen fluctuations during this period,^3^^,^^8^^,^^9^^,^^90^^,^^91^ manifested as recurrent sharp negative δ^13^C excursions superimposed on a high-δ^13^C background.

However, even after the GOE-II and the ensuing NOE, deep-water ventilation lagged, with stratified and anoxic water masses persisting (Figure 2C).^27^^,^^28^^,^^97^ Under such conditions, oxygen oases may have remained important. For instance, Fe speciation, trace-metal enrichments, and sulfur isotopes demonstrate oxygenated bottom waters within the fossil-rich layers that contain benthic macroalgae and microbial mats, whereas the adjacent non-fossil layers are indicated to deposit under anoxic conditions in the Tonian,^98^ resembling modern benthic oxygen oases in photosynthetically active, low-oxygen lakes.^59^^,^^60^ Comparable signals, including carbonate-associated Fe and trace fossils of mobile animals within microbial mats, suggest localized refugia for early oxygen-demanding metazoans in the Ediacaran.^99^^,^^100^ Altogether, during and after the GOE-II, rising atmospheric O_2_ increasingly facilitated global oceanic oxygenation, yet localized oxygen oases persisted well into the terminal Proterozoic (Figure 2C).

The final major oxygenation step (GOE-III and the associated POE) elevated atmospheric and thereafter oceanic oxygen to near-modern levels (Figures 1B and 1C). The expansion of terrestrial vegetation, especially deep-rooted vascular plants, enhanced global photosynthesis and promoted terrestrial organic carbon burial through the proliferation of land biomass.^23^^,^^24^ In addition, intensified continental weathering and nutrient (notably phosphorus) delivery to the oceans stimulated marine productivity and organic carbon burial, together boosting and stabilizing atmospheric O_2_ at higher levels,^1^^,^^3^ while facilitating pervasive deep-ocean ventilation at >420–380 Ma, as evidenced by multiple isotopic proxies (Mo, Tl, Se) and redox-sensitive elements (Mo, U, Fe, I).^14^^,^^15^^,^^16^^,^^17^^,^^20^^,^^21^ Since then, the ocean’s redox landscape has remained broadly stable, although regional and transient anoxia persisted in mid-depth waters where organic export and/or restricted circulation caused oxygen demand to exceed atmospheric supply.^101^ These anoxic zones, typically expressed as oxygen minimum zones (OMZs), waxed and waned with climatic and oceanographic fluctuations (Figure 2D). In general, elevated temperatures, stronger stratification, and enhanced productivity linked to nutrient loading could all expand OMZs, leading to oceanic anoxic events.^33^^,^^34^^,^^35^ However, unlike the long-lived, pervasive anoxia of the Precambrian oceans, Phanerozoic anoxic events were short-lived and often followed by widespread deposition of iron-rich red beds^102^^,^^103^^,^^104^—reflecting the enhanced oxygen-buffering capacity established after the GOE-III and the ensuing POE, when high atmospheric O_2_ levels and depleted oceanic reductant inventories together prevented sustained global anoxia.

In brief, from the POE to the present, the ocean has exhibited three defining redox features: an oxygen-supersaturated photic zone fueled by local oxygenic photosynthesis; a well-ventilated deep ocean, efficiently transporting atmospheric O_2_ via global circulation; and localized mid-depth OMZs along continental margins, controlled by organic export and circulation dynamics (Figure 2D).^105^ Intriguingly, modern redox-stratified lakes, such as those in Antarctica and Venezuela,^59^^,^^60^ host benthic microbial photosynthesis that sustains localized oxygen oases—rare analogs of the ecological and geochemical niches that once shaped the early Earth’s ocean oxygenation (Figure 2).

## Stagewise atmosphere-ocean oxygenation shaped life’s evolutionary tempo

Earth’s atmosphere-ocean oxygenation modulated biological evolution not as a single deterministic trigger, but as a dynamic environmental constraint whose impact depended on oxygen concentration, stability, and spatial heterogeneity. Stagewise rises in atmospheric O_2_, superimposed on persistently heterogeneous oceanic redox landscapes, shaped metabolic feasibility and ecological opportunity, yet biological responses lagged atmospheric oxygenation because of oceanic redox buffering and ecological limitations (Figures 1B and 1C).^1^^,^^2^^,^^3^ Before and during the GOE-I, efficient surface oxygen sinks confined oxygenation to episodic, shallow-water oxygen oases (Figure 2A), providing only transient ecological windows for early aerobic metabolisms.^2^ Although atmospheric O_2_ accumulation afterward fundamentally altered surface redox conditions, it did not generate persistently oxygenated oceans (Figure 2B), and evolutionary innovation remained limited, even as eukaryotes emerged.^2^^,^^6^ During and after the GOE-II, oceanic oxygen availability increased but remained spatially and temporally dynamic. Under these conditions, O_2_ acted primarily as a permissive constraint, expanding habitable niches while complex multicellularity and early animals evolved under fluctuating oxygen levels (Figure 2C).^7^^,^^10^^,^^11^^,^^100^^,^^106^ This interval likely witnessed the consolidation of oxygen-sensing and hypoxia-response pathways, enabling organisms to couple environmental oxygen gradients to gene regulation, developmental patterning, and physiological control.^107^ Although the causal role of oxygen in early animal evolution remains debated,^11^^,^^100^^,^^107^ rising oxygen levels may have expanded ecological opportunity rather than directly triggering innovation.^106^ By the GOE-III, more stable, widespread ocean oxygenation crossed key physiological thresholds, actively amplifying ecological complexity (Figure 2D). Expansion of terrestrial plant cover raised atmospheric O_2_ through intensified weathering, nutrient delivery, and organic carbon burial,^23^^,^^24^ while improved ocean ventilation supported the diversification of large, mobile, and metabolically demanding organisms. Oxygen levels on Earth’s surface were now sufficiently high, stable, and pervasive to permit persistent occupation of deeper habitats, active predation, larger body sizes, and increasingly complex food webs.^2^

Collectively, Earth’s surface oxygenation process regulated biological evolution through shifting modes of control: strongly constraining early innovation under largely anoxic Archean-early Proterozoic conditions, permissively enabling eukaryotic and early animal evolution in the redox-dynamic Proterozoic, and actively amplifying ecological complexity once stable, high-O_2_ conditions were established in the Paleozoic.

## Conclusion and perspective

Earth’s surface oxygenation history reflects a dynamic and nonlinear interplay among atmospheric O_2_ evolution, land and ocean redox buffering, and ecological innovation. From transient Archean oxygen oases in the oceans to stagewise rises in atmospheric *p*O_2_—each followed by major, yet often delayed, episodes of oceanic oxygenation—and ultimately to near-modern oxygen levels in both reservoirs, oxygen accumulation progressed unevenly through space and time. This synthesis reveals a clear first-order narrative. Free oxygen first emerged in the photic zone of surface oceans, generating localized oxygen oases by ∼3.0 Ga or earlier. Atmospheric O_2_ subsequently accumulated and rose through multiple major steps beginning at ∼2.4 Ga, driven initially by biological oxygen production in surface oceans and later augmented by terrestrial ecosystems. Each step promoted progressively more extensive—but temporally lagged—ventilation of the deep ocean. Fully sustained oxygenation of the ocean interior was not achieved until ∼0.41 Ga, when atmospheric O_2_ approached near-modern levels. This evolutionary pathway defines a characteristic sequence of Earth’s surface oxygenation: ocean → atmosphere → ocean. Throughout the 2-billion-year oxygenation transition, atmospheric and oceanic oxygen levels were episodically decoupled on shorter timescales, superimposed on a long-term trajectory toward global oxidation. Ocean redox evolution was further modulated by local productivity, reductant fluxes, and basin paleogeography, producing spatiotemporally heterogeneous redox landscapes that alternately enabled and constrained biological innovation. Overall, the trajectory from localized Archean oxygen oases to the globally well-oxygenated surface environments of today underscores how atmosphere-ocean oxygenation both governs—and is reciprocally shaped by—the evolution of life.

Looking ahead, advancing our understanding of Earth’s oxygenation history—and informing the search for other habitable worlds—will benefit from several key directions. (1) High-resolution reconstruction of atmospheric O_2_: improved calibration of atmospheric O_2_ proxies, particularly through expanded sulfate Δ′^17^O records integrated with refined geochronology and development of quantitative models linking sulfate Δ′^17^O to atmospheric O_2_ levels, will more tightly constrain the timing and tempo of atmospheric oxygenation. (2) Multi-proxy, spatially resolved approaches: integrating diverse geological proxies across spatial and temporal scales is essential for disentangling local from global redox signals and capturing the full complexity of oceanic oxygenation. (3) Coupled tectonic-biogeochemical modeling: linking tectonic and volcanic activity with atmospheric-oceanic oxygen evolution and biological innovation will help reveal the underlying mechanisms driving each major oxygenation step. (4) Interdisciplinary integration: combining surface redox records with palaeobiological, modern, and planetary perspectives can illuminate how oxygen availability shaped ecological niches, evolutionary thresholds, and planetary habitability.

## Funding and acknowledgments

Financial support was supported by the 10.13039/501100012166National Key Research and Development Program of China (grant no. 2022YFF0800100 to C.L.) and the 10.13039/501100001809National Natural Science Foundation of China (grant nos. 42425002 to C L. and 42572129 and 42103072 to H.W.). The funders had no role in study design, data collection and analysis, decision to publish, or preparation of the manuscript.

## Author contributions

H.W. and C.L. supervised the review, and wrote and revised the manuscript. Both authors contributed to the manuscript and approved the final version.

## Declaration of interests

The authors declare no competing interests.

## Supplemental information

Document S1. Figure S1

Document S2. Article plus supplemental information

## References

[1] Lyons T.W., Tino C.J., Fournier G.P. (2024). Co-evolution of early Earth environments and microbial life. Nat. Rev. Microbiol..

[2] Knoll A.H., Nowak M.A. (2017). The timetable of evolution. Sci. Adv..

[3] Wang H., Li C., Peng Y. (2025). Two-billion-year transitional oxygenation of the Earth’s surface. Nature.

[4] Catling D.C., Zahnle K.J. (2020). The Archean atmosphere. Sci. Adv..

[5] Farquhar J., Bao H., Thiemens M. (2000). Atmospheric Influence of Earth’s Earliest Sulfur Cycle. Science.

[6] Butterfield N.J. (2015). Early evolution of the Eukaryota. Palaeontology.

[7] Tang Q., Zheng W., Zhang S. (2024). Quantifying the global biodiversity of Proterozoic eukaryotes. Science.

[8] Och L.M., Shields-Zhou G.A. (2012). The Neoproterozoic oxygenation event: Environmental perturbations and biogeochemical cycling. Earth. Sci. Rev..

[9] Krause A.J., Mills B.J.W., Merdith A.S. (2022). Extreme variability in atmospheric oxygen levels in the late Precambrian. Sci. Adv..

[10] Darroch S.A.F., Smith E.F., Laflamme M. (2018). Ediacaran Extinction and Cambrian Explosion. Trends Ecol. Evol..

[11] Evans S.D., Diamond C.W., Droser M.L. (2018). Dynamic oxygen and coupled biological and ecological innovation during the second wave of the Ediacara Biota. Emerg. Top. Life Sci..

[12] Wang H., Peng Y., Li C. (2023). Sulfate triple-oxygen-isotope evidence confirming oceanic oxygenation 570 million years ago. Nat. Commun..

[13] Krause A.J., Mills B.J.W., Zhang S. (2018). Stepwise oxygenation of the Paleozoic atmosphere. Nat. Commun..

[14] Dahl T.W., Hammarlund E.U., Anbar A.D. (2010). Devonian rise in atmospheric oxygen correlated to the radiations of terrestrial plants and large predatory fish. Proc. Natl. Acad. Sci. USA.

[15] Wallace M.W., Hood A.v., Shuster A. (2017). Oxygenation history of the Neoproterozoic to early Phanerozoic and the rise of land plants. Earth Planet Sci. Lett..

[16] Stolper D.A., Keller C.B. (2018). A record of deep-ocean dissolved O_2_ from the oxidation state of iron in submarine basalts. Nature.

[17] Stockey R.G., Cole D.B., Farrell U.C. (2024). Sustained increases in atmospheric oxygen and marine productivity in the Neoproterozoic and Palaeozoic eras. Nat. Geosci..

[18] Scott A.C., Glasspool I.J. (2006). The diversification of Paleozoic fire systems and fluctuations in atmospheric oxygen concentration. Proc. Natl. Acad. Sci. USA.

[19] Glasspool I.J., Gastaldo R.A. (2022). Silurian wildfire proxies and atmospheric oxygen. Geology.

[20] Bubphamanee K., Kipp M.A., Meixnerová J. (2025). Mid-Devonian ocean oxygenation enabled the expansion of animals into deeper-water habitats. Proc. Natl. Acad. Sci. USA.

[21] Ostrander C.M., Clemente J.N.R., Stockey R.G. (2025). Dynamic deep marine oxygenation during the Early and Middle Paleozoic. Sci. Adv..

[22] Zhang J., Edwards C.T., Diamond C.W. (2021). Marine oxygenation, deoxygenation, and life during the Early Paleozoic: An overview. Palaeogeogr. Palaeoclimatol. Palaeoecol..

[23] Waldeck A.R., Olson H.C., Crockford P.W. (2025). Marine sulphate captures a Paleozoic transition to a modern terrestrial weathering environment. Nat. Commun..

[24] Yuan W., Liu M., Chen D. (2023). Mercury isotopes show vascular plants had colonized land extensively by the early Silurian. Sci. Adv..

[25] Dodd M.S., Wang H., Li C. (2022). Abiotic anoxic iron oxidation, formation of Archean banded iron formations, and the oxidation of early Earth. Earth Planet Sci. Lett..

[26] Bekker A., Slack J.F., Planavsky N. (2010). Iron Formation: The Sedimentary Product of a Complex Interplay among Mantle, Tectonic, Oceanic, and Biospheric Processes. Econ. Geol..

[27] Li C., Love G.D., Lyons T.W. (2010). A Stratified Redox Model for the Ediacaran Ocean. Science.

[28] Canfield D.E., Poulton S.W., Knoll A.H. (2008). Ferruginous Conditions Dominated Later Neoproterozoic Deep-Water Chemistry. Science.

[29] Poulton S.W., Canfield D.E. (2011). Ferruginous Conditions: A Dominant Feature of the Ocean through Earth’s History. Elements.

[30] Wang H., Li C., Cheng M. (2022). Redbed formation in the redox-stratified mid-Proterozoic ocean. Precambr. Res..

[31] Wang H., Li C., Hu C. (2015). Spurious thermoluminescence characteristics of the Ediacaran Doushantuo Formation (ca. 635–551 Ma) and its implications for marine dissolved organic carbon reservoir. J. Earth Sci..

[32] Remírez M.N., Algeo T.J. (2020). Carbon-cycle changes during the Toarcian (Early Jurassic) and implications for regional versus global drivers of the Toarcian oceanic anoxic event. Earth Sci. Rev..

[33] Erbacher J., Huber B.T., Norris R.D. (2001). Increased thermohaline stratification as a possible cause for an ocean anoxic event in the Cretaceous period. Nature.

[34] Kunert A., Kendall B. (2023). Global ocean redox changes before and during the Toarcian Oceanic Anoxic Event. Nat. Commun..

[35] Yao W., Kong T., Wang X.T. (2024). Expanded subsurface ocean anoxia in the Atlantic during the Paleocene-Eocene Thermal Maximum. Nat. Commun..

[36] Planavsky N.J., Reinhard C.T., Isson T.T. (2020). Large Mass-Independent Oxygen Isotope Fractionations in Mid-Proterozoic Sediments: Evidence for a Low-Oxygen Atmosphere?. Astrobiology.

[37] Li C., Wang H. (2025). Earth’s atmosphere took two billion years to become fully oxygenated. Nature.

[38] Wang H., Li C., Peng Y. (2026). Evolution of a habitable Earth: tracing Earth’s oxygenation pathway through triple oxygen isotopes (in Chinese). Chin. Sci. Bull..

[39] Bao H. (2015). Sulfate: A time capsule for Earth’s O_2_, O_3_, and H_2_O. Chem. Geol..

[40] Cao X., Bao H. (2013). Dynamic model constraints on oxygen-17 depletion in atmospheric O_2_ after a snowball Earth. Proc. Natl. Acad. Sci. USA.

[41] Turner E.C., Bekker A. (2015). Thick sulfate evaporite accumulations marking a mid-Neoproterozoic oxygenation event (Ten Stone Formation, Northwest Territories, Canada). Geol. Soc. Am. Bull..

[42] Pogge von Strandmann P.A.E., Stüeken E.E., Elliott T. (2015). Selenium isotope evidence for progressive oxidation of the Neoproterozoic biosphere. Nat. Commun..

[43] Qin Z., Xu D., Kendall B. (2022). Molybdenum isotope-based redox deviation driven by continental margin euxinia during the early Cambrian. Geochim. Cosmochim. Acta.

[44] Zhang F., Stockey R.G., Xiao S. (2022). Uranium isotope evidence for extensive shallow water anoxia in the early Tonian oceans. Earth Planet Sci. Lett..

[45] Lu W., Ridgwell A., Thomas E. (2018). Late inception of a resiliently oxygenated upper ocean. Science.

[46] Liu X.-M., Kah L.C., Knoll A.H. (2016). Tracing Earth’s O_2_ evolution using Zn/Fe ratios in marine carbonates. Geochem. Perspect. Lett..

[47] Scott C., Lyons T.W., Bekker A. (2008). Tracing the stepwise oxygenation of the Proterozoic ocean. Nature.

[48] Lu W., Wörndle S., Halverson G.P. (2017). Iodine proxy evidence for increased ocean oxygenation during the Bitter Springs Anomaly. Geochem. Perspect. Lett..

[49] Sahoo S.K., Planavsky N.J., Jiang G. (2016). Oceanic oxygenation events in the anoxic Ediacaran ocean. Geobiology.

[50] Partin C.A., Bekker A., Planavsky N.J. (2013). Large-scale fluctuations in Precambrian atmospheric and oceanic oxygen levels from the record of U in shales. Earth Planet Sci. Lett..

[51] Reinhard C.T., Planavsky N.J. (2022). The History of Ocean Oxygenation. Ann. Rev. Mar. Sci.

[52] Wang H., Zhang Z., Li C. (2020). Spatiotemporal redox heterogeneity and transient marine shelf oxygenation in the Mesoproterozoic ocean. Geochim. Cosmochim. Acta.

[53] Reinhard C.T., Planavsky N.J., Olson S.L. (2016). Earth’s oxygen cycle and the evolution of animal life. Proc. Natl. Acad. Sci. USA.

[54] He R., Pohl A., Zhang X. (2026). A reversed latitudinal ocean oxygen gradient in the Proterozoic Eon. Nat. Geosci..

[55] Canfield D.E., Zhang S., Frank A.B. (2018). Highly fractionated chromium isotopes in Mesoproterozoic-aged shales and atmospheric oxygen. Nat. Commun..

[56] Kump L.R. (2014). Hypothesized link between Neoproterozoic greening of the land surface and the establishment of an oxygen-rich atmosphere. Proc. Natl. Acad. Sci. USA.

[57] Fike D.A., Grotzinger J.P., Pratt L.M. (2006). Oxidation of the Ediacaran Ocean. Nature.

[58] Dodd M.S., Li C., Gu H. (2025). Marine phosphorus and atmospheric oxygen were coupled during the Great Oxidation Event. Nat. Commun..

[59] Gingras M., Hagadorn J.W., Seilacher A. (2011). Possible evolution of mobile animals in association with microbial mats. Nat. Geosci..

[60] Sumner D.Y., Hawes I., Mackey T.J. (2015). Antarctic microbial mats: A modern analog for Archean lacustrine oxygen oases. Geology.

[61] Conley D.J., Carstensen J., Aigars J. (2011). Hypoxia is increasing in the coastal zone of the baltic sea. Environ. Sci. Technol..

[62] Queste B.Y., Fernand L., Jickells T.D. (2013). Spatial extent and historical context of North Sea oxygen depletion in August 2010. Biogeochemistry.

[63] Fischer W.W., Hemp J., Johnson J.E. (2016). Evolution of Oxygenic Photosynthesis. Annu. Rev. Earth Planet Sci..

[64] Satkoski A.M., Beukes N.J., Li W. (2015). A redox-stratified ocean 3.2 billion years ago. Earth Planet Sci. Lett..

[65] Freimann M.A., Knauer L.G., Kuchenbecker M. (2021). New geochronologic and geochemical constraints for the Pedro Pereira metavolcanosedimentary sequence: Evidence for a 2.77 Ga oxygen oasis record in the São Francisco-Congo paleocontinent. J. J. South Am. Earth Sci..

[66] Ossa Ossa F., Hofmann A., Spangenberg J.E. (2019). Limited oxygen production in the Mesoarchean ocean. Proc. Natl. Acad. Sci. USA.

[67] Eickmann B., Hofmann A., Wille M. (2018). Isotopic evidence for oxygenated Mesoarchaean shallow oceans. Nat. Geosci..

[68] Kaufman A.J., Johnston D.T., Farquhar J. (2007). Late Archean Biospheric Oxygenation and Atmospheric Evolution. Science.

[69] Ye H., Wu C.-Z., Wang X.-L. (2025). Proliferation of oxygen oases in Mesoarchean oceans. Geology.

[70] Kurzweil F., Wille M., Gantert N. (2016). Manganese oxide shuttling in pre-GOE oceans–evidence from molybdenum and iron isotopes. Earth Planet Sci. Lett..

[71] Chen X., Ostrander C.M., Holdaway B.J. (2025). Transient marine bottom water oxygenation on continental shelves by 2.65 billion years ago. Nat. Geosci..

[72] Ostrander C.M., Nielsen S.G., Owens J.D. (2019). Fully oxygenated water columns over continental shelves before the Great Oxidation Event. Nat. Geosci..

[73] Anbar A.D., Duan Y., Lyons T.W. (2007). A Whiff of Oxygen Before the Great Oxidation Event?. Science.

[74] Liang X., Stüeken E.E., Alessi D.S. (2025). A seawater oxygen oscillation recorded by iron formations prior to the Great Oxidation Event. Nat. Geosci..

[75] Cañadas F., Guilbaud R., Fralick P. (2025). Archaean oxygen oases driven by pulses of enhanced phosphorus recycling in the ocean. Nat. Geosci..

[76] Kendall B., Reinhard C.T., Lyons T.W. (2010). Pervasive oxygenation along late Archaean ocean margins. Nat. Geosci..

[77] Holland H.D. (2006). The oxygenation of the atmosphere and oceans. Philos. Trans. R. Soc. Lond. B Biol. Sci..

[78] Olson S.L., Kump L.R., Kasting J.F. (2013). Quantifying the areal extent and dissolved oxygen concentrations of Archean oxygen oases. Chem. Geol..

[79] Stüeken E.E., Kipp M.A., Koehler M.C. (2016). The evolution of Earth’s biogeochemical nitrogen cycle. Earth Sci. Rev..

[80] Ostrander C.M., Heard A.W., Shu Y. (2024). Onset of coupled atmosphere–ocean oxygenation 2.3 billion years ago. Nature.

[81] Heard A.W., Ostrander C.M., Shu Y. (2025). Onset of persistent surface ocean oxygenation during the Great Oxidation Event. Nat. Commun..

[82] Tang D., Shi X., Wang X. (2016). Extremely low oxygen concentration in mid-Proterozoic shallow seawaters. Precambr. Res..

[83] Wang C., Lechte M.A., Reinhard C.T. (2022). Strong evidence for a weakly oxygenated ocean–atmosphere system during the Proterozoic. Proc. Natl. Acad. Sci. USA.

[84] Liu X.M., Kah L.C., Knoll A.H. (2021). A persistently low level of atmospheric oxygen in Earth’s middle age. Nat. Commun..

[85] Zhao C., Shi M., Feng Q. (2020). New study of microbial mats from the Mesoproterozoic Jixian Group, North China: Evidence for photosynthesis and oxygen release. Precambr. Res..

[86] Jia T., Wang R., Huang T. (2022). Constraining the redox landscape of Mesoproterozoic mat grounds: A possible oxygen oasis in the ‘Boring Billion’ seafloor. Precambr. Res..

[87] Xu L., Shi X., Poulton S.W. (2025). Shallow seawater oxygenation at ca. 1.44 Ga: A reflection of local seafloor oxygen oases or extensive water-column oxygenation?. Geol. Soc. Am. Bull..

[88] Dodd M.S., Shi W., Li C. (2023). Uncovering the Ediacaran phosphorus cycle. Nature.

[89] Reinhard C.T., Planavsky N.J., Gill B.C. (2017). Evolution of the global phosphorus cycle. Nature.

[90] Canfield D.E., Knoll A.H., Poulton S.W. (2020). Carbon isotopes in clastic rocks and the Neoproterozoic carbon cycle. Am. J. Sci..

[91] Mitchell R.N., Feng L., Zhang Z. (2023). Carbonate-organic decoupling during the first Neoproterozoic carbon isotope excursion. Innov. Geosci..

[92] Cole D.B., Reinhard C.T., Wang X. (2016). A shale-hosted Cr isotope record of low atmospheric oxygen during the Proterozoic. Geology.

[93] Zhang F., Xiao S., Romaniello S.J. (2019). Global marine redox changes drove the rise and fall of the Ediacara biota. Geobiology.

[94] Chen B., Hu C., Mills B.J.W. (2022). A short-lived oxidation event during the early Ediacaran and delayed oxygenation of the Proterozoic ocean. Earth Planet Sci. Lett..

[95] Kunzmann M., Bui T.H., Crockford P.W. (2017). Bacterial sulfur disproportionation constrains timing of neoproterozoic oxygenation. Geology.

[96] Li C., Wang H. (2024). Uncovering the largest negative carbon isotope excursion in Earth history. Sci. China Earth Sci..

[97] Sperling E.a., Wolock C.J., Morgan A.S. (2015). Statistical analysis of iron geochemical data suggests limited late Proterozoic oxygenation. Nature.

[98] Wang H., Liu A., Li C. (2021). A benthic oxygen oasis in the early Neoproterozoic ocean. Precambr. Res..

[99] Ding W., Dong L., Sun Y. (2019). Early animal evolution and highly oxygenated seafloor niches hosted by microbial mats. Sci. Rep..

[100] Xiao S., Chen Z., Zhou C. (2019). Surfing in and on microbial mats: Oxygen-related behavior of a terminal Ediacaran bilaterian animal. Geology.

[101] Wang X., Algeo T.J., Li C. (2023). Spatial pattern of marine oxygenation set by tectonic and ecological drivers over the Phanerozoic. Nat. Geosci..

[102] Hu X., Scott R.W., Cai Y. (2012). Cretaceous oceanic red beds (CORBs): Different time scales and models of origin. Earth Sci. Rev..

[103] Wang C., Hu X., Huang Y. (2011). Cretaceous oceanic red beds as possible consequence of oceanic anoxic events. Sediment. Geol..

[104] Song H., Jiang G., Poulton S.W. (2017). The onset of widespread marine red beds and the evolution of ferruginous oceans. Nat. Commun..

[105] Paulmier A., Ruiz-Pino D. (2009). Oxygen minimum zones (OMZs) in the modern ocean. Prog. Oceanogr..

[106] Li C., Jin C., Planavsky N.J. (2017). Coupled oceanic oxygenation and metazoan diversification during the early–middle Cambrian?. Geology.

[107] Hammarlund E.U., Flashman E., Mohlin S. (2020). Oxygen-sensing mechanisms across eukaryotic kingdoms and their roles in complex multicellularity. Science.

