# Supplementary material for: Stagewise oxygenation of the Earth’s atmosphere and oceans
Source: Innovation (Camb). 2026 Mar 17;7(9):101353. doi: 10.1016/j.xinn.2026.101353 (PMC13556354; doi:10.1016/j.xinn.2026.101353)
Supplement: Supplementary file 1 [file mmc1.pdf]

The Innovation, Volume *7*

Supplemental Information

Stagewise oxygenation of the Earth's atmosphere and oceans

Haiyang Wang and Chao Li

SUPPLEMENTARY TEXT

Oxygen isotope notation

$\delta'^{18}O = \ln{}^{18}R \times 1000‰$, $\delta'^{17}O = \ln{}^{17}R \times 1000‰$, $\Delta'^{17}O = \delta'^{17}O - 0.5305 \times \delta'^{18}O$, where $^{18}R = (^{18}O/^{16}O)_{sample} / (^{18}O/^{16}O)_{standard}$, $^{17}R = (^{17}O/^{16}O)_{sample} / (^{17}O/^{16}O)_{standard}$. The standard refers to Vienna Standard Mean Ocean Water.

Origin of atmospheric O-MIF

Oxygen mass-independent fractionation (O-MIF) in atmospheric O_2 arises from photochemical O_2-O_3-CO_2 reactions in the stratosphere. These reactions enrich ^{17}O in O_3 ($\Delta'^{17}O > 0$), transfer a positive ^{17}O anomaly to CO_2 ($\Delta'^{17}O > 0$), and leave residual atmospheric O_2 depleted in ^{17}O ($\Delta'^{17}O < 0$; Figure S1A).[1] The magnitude of $\Delta'^{17}O$ in atmospheric O_2 is controlled by three interacting factors: (i) atmospheric pCO_2, which sequesters positive ^{17}O anomalies; (ii) gross primary productivity (GPP), which supplies O_2 with near-zero $\Delta'^{17}O$ and dilutes the O-MIF signal; and (iii) the size of the atmospheric O_2 reservoir (pO_2). These controls are captured by the ratios pO_2/pCO_2 and pO_2/GPP (that is, the O_2 residence time), with higher pO_2/GPP and/or lower pO_2/pCO_2 ratios generally yielding more negative $\Delta'^{17}O$ values in atmospheric O_2 (Figure S1B).[2,3]

Transfer and preservation of O-MIF in sedimentary sulfate

Atmospheric O-MIF signals can be transferred to sulfate during the oxidation of reduced sulfur species by atmospheric O_2 and preserved in sedimentary sulfate phases, including gypsum ($CaSO_4$), barite ($BaSO_4$), and carbonate-associated sulfate (CAS), yielding negative sulfate $\Delta'^{17}O$ values.[1] Reduced sulfur sources include terrestrial pyrite and marine H_2S/HS^-/dissolved organic sulfur, linking sulfate $\Delta'^{17}O$ signatures to continental weathering intensity and/or oceanic oxygenation events (Figure S1A).[3,4] Once incorporated into sulfate, subsequent processes—including microbial sulfur cycling and metamorphic overprinting—can only attenuate the primary O-MIF signal, driving sulfate $\Delta'^{17}O$ values toward 0‰. The recurring occurrence of strongly ^{17}O-depleted sulfate in ancient sediments, in some cases exhibiting $\Delta'^{17}O$ values even lower than modern atmospheric O_2 ($\Delta'^{17}O \approx -0.5‰$), therefore implies substantially larger atmospheric O-MIF signatures than today.[3] Such signals point to fundamentally different atmospheric chemistry, consistent with lower pO_2/pCO_2 and/or higher pO_2/GPP ratios.[2] With independent constraints on pCO_2 and GPP, or through integrated biogeochemical models linking pO_2, pCO_2, and GPP, sulfate $\Delta'^{17}O$ can be used to quantitatively constrain atmospheric O_2 levels.[3] Three major

shifts in sulfate $\Delta'^{17}O$ associated with GOE-I, GOE-II, and GOE-III record a long-term, stagewise rise in atmospheric oxygen from pre-GOE-I near-zero conditions ($<10^{-6}$ PAL; PAL: the present atmospheric level) to near-modern levels (~1 PAL; Figure S1B).[3]

SUPPLEMENTARY FIGURE

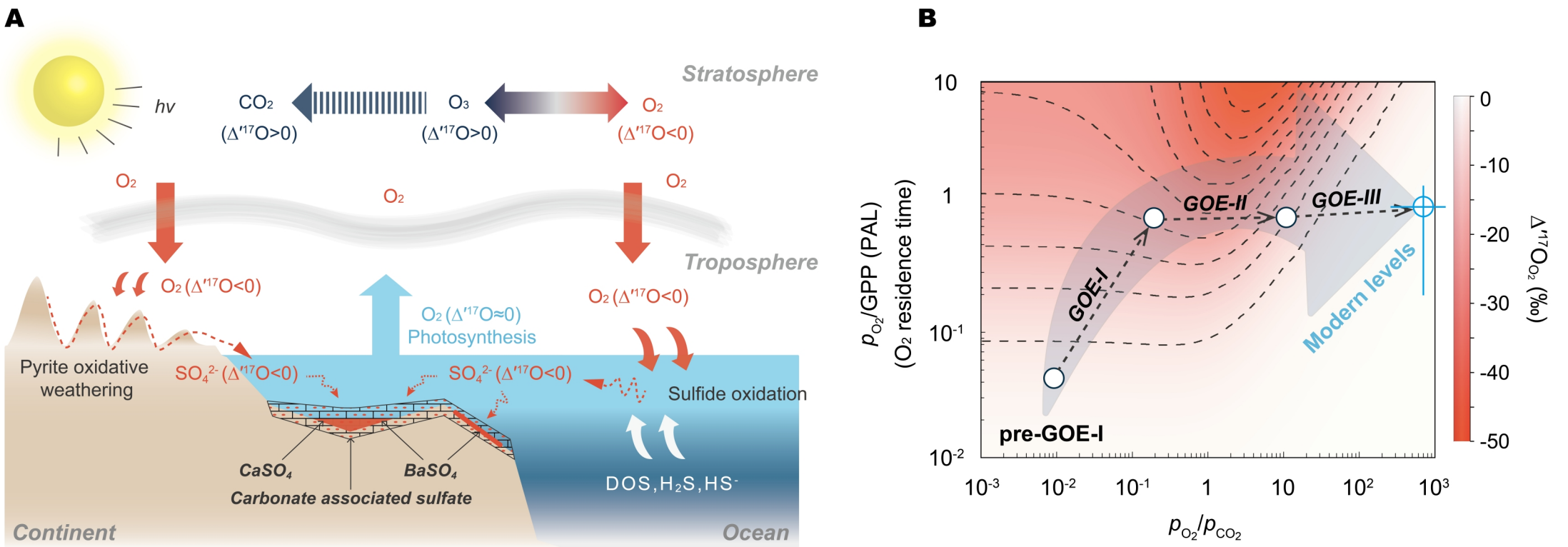

Figure S1. Atmospheric O-MIF and the sulfate $\Delta'^{17}O$ proxy for atmospheric O_2 (modified from Wang et al.[3]). **(A)** Mass-independent ^{17}O-depleted O_2 is produced in the stratosphere, which is incorporated into sulfate via reduced-sulfur oxidation. **(B)** At steady state, $\Delta'^{17}O$ (O_2) correlates with the pO_2/pCO_2 and pO_2/GPP ratios.

REFERENCES

1. Bao, H. (2015). Sulfate: A time capsule for Earth's O_2, O_3, and H_2O. *Chem. Geol.* **395**: 108–118. DOI: 10.1016/j.chemgeo.2014.11.025
2. Cao, X., and Bao, H. (2013). Dynamic model constraints on oxygen-17 depletion in atmospheric O_2 after a snowball Earth. *Proc. Natl. Acad. Sci. U.S.A.* **110**: 14546–14550. DOI: 10.1073/pnas.1302972110
3. Wang, H., Li, C., Peng, Y. et al. (2025). Two-billion-year transitional oxygenation of the Earth's surface. *Nature* **645**: 665–671. DOI: 10.1038/s41586-025-09471-4
4. Wang, H., Peng, Y., Li, C. et al. (2023). Sulfate triple-oxygen-isotope evidence confirming oceanic oxygenation 570 million years ago. *Nat. Commun.* **14**: 4315. DOI: 10.1038/s41467-023-39962-9
